# Clinical, Cognitive and Demographic Determinants of Work Participation in Multiple Sclerosis: A Multicenter Cross-Sectional Study

**DOI:** 10.3390/medicina62030454

**Published:** 2026-02-27

**Authors:** Konstantina Stavrogianni, Dimitrios K. Kitsos, Evangelia-Makrina Dimitriadou, Alexandra Akrivaki, Athanasios K. Chasiotis, Pinelopi Vlotinou, George P. Paraskevas, Georgios Tsivgoulis, Daphne Bakalidou, Konstantinos Tsamis, Dimitrios Peschos, Vasileios Giannopapas, John S. Tzartos, Sotirios Giannopoulos

**Affiliations:** 1 Second Department of Neurology, National and Kapodistrian University of Athens, 12462 Athens, Greece; stavrogianni.k@gmail.com (K.S.); dkitsos@icloud.com (D.K.K.); evadim93@hotmail.gr (E.-M.D.); alexandra.akrivaki@gmail.com (A.A.); thanosch1@gmail.com (A.K.C.); geoprskvs44@gmail.com (G.P.P.); tsivgoulisgiorg@yahoo.gr (G.T.); bgiannopapas@gmail.com (V.G.); jtzartos@gmail.com (J.S.T.); 2 Department of Physiology, Faculty of Medicine, School of Health Sciences, University of Ioannina, 45110 Ioannina, Greecedpeschos@uoi.gr (D.P.); 3 Interdisciplinary Laboratoy of Research, Education and Disability Support (IREDS Lab), Department of Physiotherapy, Faculty of Health and Caring Professions, University of West Attica, 12243 Attica, Greece; dafbak@otenet.gr; 4 Occupational Therapy Department, University of West Attica, 12243 Attica, Greece; pvlotinou@uniwa.gr

**Keywords:** multiple sclerosis, employment status, disability, lower urinary tract symptoms (LUTS), information processing speed, fatigue, quality of life

## Abstract

*Background and Objectives*: Employment is a major determinant of quality of life in people with multiple sclerosis (pwMS). This multicenter cross-sectional study aimed to identify which commonly studied demographic, disease-related, clinical, cognitive, and psychological variables, alongside the presence of lower urinary tract symptoms (LUTS), predict employment status in pwMS. *Materials and Methods*: Seventy-eight pwMS were classified as either full-time employed (*n* = 41) or non-employed (*n* = 37). Participants underwent clinical and neuropsychological assessment including disability status (Expanded Disability Status Scale; EDSS), fatigue (Modified Fatigue Impact Scale; MFIS), information processing speed (Symbol Digit Modalities Test; SDMT), depressive symptoms (Hospital Anxiety and Depression Scale-Depression; HADS-D), and LUTS status (presence/absence), alongside demographic and disease-related variables (sex, age, education level, relationship status, and disease duration). *Results*: Hierarchical binary logistic regression indicated that higher information processing speed was associated with higher odds of employment (OR = 1.11, *p* = 0.008), whereas the presence of LUTS was associated with lower odds of employment (OR = 0.13, *p* = 0.026). Disability severity, fatigue, depressive symptoms, demographic characteristics, and disease duration did not contribute in the final model (*p* > 0.05). *Conclusions*: Information processing speed and urinary dysfunction were associated with employment status in pwMS. Within the present sample, the multivariable model including these variables showed good discrimination between employed and non-employed participants. The findings should be interpreted as exploratory, and they require further confirmation in independent cohorts before any potential application is considered.

## 1. Introduction

Multiple sclerosis (MS) is a chronic autoimmune disorder of the central nervous system (CNS), characterized by demyelination and axonal injury driven by both inflammatory and neurodegenerative processes [1]. MS affects approximately 2.9 million individuals globally [2] and is most frequently diagnosed between the ages of 20 and 50 years [3], typically coinciding with peak workforce participation and broader socioeconomic productivity. It occurs more commonly in women than in men and represents the leading non-traumatic cause of disability among young adults [3], due to its wide range of clinical symptoms and manifestations.

Beyond its neurological manifestations, MS frequently affects domains that are central to everyday functioning and social participation, including physical functioning, energy levels, cognition, and emotional burden [4]. Employment is one of the most meaningful indicators of participation in adult life, closely linked to autonomy, social identity, well-being and quality of life. However, maintaining work can be challenging for people with MS (pwMS), as symptoms fluctuate, disability can accumulate over time, and “invisible” determinants such as fatigue and cognitive slowing may compromise efficiency and endurance even when physical function appears relatively preserved [5]. As a result, unemployment and work instability are common outcomes in MS and are associated with downstream consequences for mental health, social inclusion, and health-related quality of life [6].

Work ability in MS is multifactorial and reflects the combined influence of neurological impairment, symptom burden, psychological functioning, and contextual factors. Physical disability in MS has been associated with poorer work outcomes, including lower work participation, greater workplace difficulties, more frequent short- and long-term sickness absence, and higher rates of unemployment. Likewise, longer disease duration has been linked to, and is often considered a contributor to, an increased risk of unemployment [7]. Cognitive dysfunction, especially reduced processing speed, memory, and executive efficiency, which is highly prevalent in MS, may also directly affect job performance in ways that are independent of physical disability and other prone manifestations of the disease [8]. Fatigue, another core symptom of MS, that is often described by patients as one of the most disabling aspects of the disease, has also been associated not only with work loss, but with the lowering of work productivity [9]. Similarly, psychological aspects (e.g., anxiety and depressive symptoms) can influence work participation through reduced motivation, concentration difficulties, and lowered stress tolerance, thus working as predictors of a higher probability of quitting a job [7].

Demographic and other related social variables may also shape employment outcomes in MS. Research highlighted MS- and health-related factors in relation to work, along with the importance of socio-demographic variables [10]. Age is often correlated with disability accumulation and comorbidity burden [11], thus making pwMS more prone towards work limitation. Furthermore, education level can serve as a proxy for cognitive reserve, occupational complexity, and access to less physically demanding work [12]. Moreover, marital or relationship status may relate to social support [13] and financial buffering, potentially influencing decisions around employment. Finally, sex-related differences in disease progression [14] and in occupational trajectories and caregiving responsibilities may further contribute to variability in work outcomes.

Urinary dysfunction is highly prevalent in MS, and general lower urinary tract symptoms (LUTS) have been reported in up to 68.4% of patients [15] and may represent an additional, often overlooked, contributor to work participation. LUTS (e.g., urgency, frequency, nocturia, and incomplete emptying) can disrupt sleep, increase fatigue and distress [16], and may create practical barriers in occupational settings where flexibility and immediate bathroom access are limited. Acknowledging urinary dysfunction as a common and clinically relevant manifestation of MS, we additionally included its presence alongside disability, cognition, fatigue, and mood to provide a more comprehensive account of the factors associated with employment status in pwMS.

In practice, these factors are likely to interact, making it useful to consider them within a unified framework rather than in isolation. Prior work has described a range of demographic, clinical, cognitive, psychological, and symptom-related correlates of employment in MS [17]; however, their relative contribution often varies across samples and methods, and single indicators may not fully reflect the broader configuration of influences associated with work participation. Accordingly, there is value in integrative models that examine multiple commonly assessed variables simultaneously, to clarify which factors show the most robust associations with employment status and to support a more structured clinical discussion of vocational needs and potential support options (e.g., symptom management, rehabilitation input, or workplace accommodations) when indicated.

Against this background, the present study examined employment status in a sample of pwMS by integrating demographic characteristics, disease-related variables, and patient-reported and objective indicators of symptom burden and cognitive efficiency. The primary aim was to determine which factors predicted employment status when considered simultaneously, focusing on variables that have been systematically examined in the literature and have been shown, to varying degrees, to be associated with and to predict work participation in pwMS. In addition to these established predictors, we also included urinary dysfunction presence as an additional variable of interest, as, to the best of our knowledge, this domain has received limited attention in multivariable models of employment status in pwMS and has not been examined as a potential independent contributor within a unified framework. A secondary aim was to descriptively examine the model-estimated probabilities using ROC analysis in order to summarize apparent discrimination within the present sample.

## 2. Materials and Methods

### 2.1. Study Design

This was a multicenter, cross-sectional study designed to examine clinical, cognitive, psychological, and demographic determinants of employment status in pwMS. Specifically, we assessed the extent to which demographic variables (age, sex, education level, and relationship status), disease-related characteristics (disease duration, disability status, presence or absence of LUTS), fatigue, cognitive processing speed, and depressive symptoms were associated with work status (employed vs. unemployed). A hierarchical binary logistic regression was used to examine the independent contribution of each predictor to occupation status. Model discrimination was evaluated using ROC analysis, and candidate probability cut-offs were reported as exploratory indices.

### 2.2. Participants

An a priori power analysis was conducted in G*Power (v3.1.9.4) [18] for binary logistic regression (z test) assuming a two-tailed α = 0.05 and 80% power. Given the established clinical relevance of disability severity for work participation in pwMS [9], the analysis targeted detection of a moderately large association for disability status (odds ratio [OR] = 2.5 for a one standard deviation increase in disability status). The baseline probability of the outcome under H0 was set to 0.50, consistent with the approximate outcome prevalence in the present sample, and the shared variance between disability status and other covariates was conservatively accounted for by specifying R^2^ other X = 0.30. This yielded a required sample size of *N* = 76; therefore, the available sample (*N* = 78) was considered adequate for detecting effects of this magnitude.

Participants were recruited from three centers in Greece: the Demyelination and Other Auto-Immune Diseases Outpatient Clinic of the Second Department of Neurology in Athens, the corresponding Outpatient Neurology service in Ioannina, and the Interdisciplinary Laboratoy of Research, Education and Disability Support (IREDS Lab). Eligible participants were required to have a definite diagnosis of MS according to the revised 2017 McDonald criteria [19], be between 18 and 59 years of age, score above 26 on the Montreal Cognitive Assessment (MoCA) [20]; a MoCA score > 26 was required as a global cognitive screening criterion to minimize the inclusion of participants with possible generalized cognitive impairment, thereby reducing confounding from global cognitive decline; and be receiving disease-modifying treatment at the time of recruitment.

Participants were excluded if they had any neurological condition other than MS, a psychiatric or neurodevelopmental disorder based on medical history and clinical records, or any comorbid medical condition known to substantially contribute to fatigue or cognitive impairment (e.g., thyroid disease, anemia, severe sleep disorder, or uncontrolled systemic disease). Pregnancy and the postpartum period were also exclusionary. In addition, participants were excluded if they had motor deficits in their dominant upper limb that prevented completion of written assessments. Finally, participants were also excluded if they were employed exclusively on a part-time basis, as we aimed to maintain a clear dichotomization of employment status and reduce heterogeneity.

### 2.3. Measures

The variables assessed included demographic factors of age (in years), relationship status (single or in a long-term relationship), and employment status (employed in full-time job or unemployed), obtained through the clinical interview.

Disease-related measures comprised disease duration (in years) and disability status evaluated with the Expanded Disability Status Scale (EDSS) [21]. LUTS were operationalized as a dichotomous clinical indicator (presence vs. absence) based on patient reports during the routine clinical documentation. LUTS was used to capture the presence of clinically relevant urinary symptomatology rather than symptom severity. No cases had documented evidence suggestive of acute urinary tract infection at the time of assessment. Fatigue was assessed using the Modified Fatigue Impact Scale (MFIS), a validated self-report questionnaire, which evaluates both physical and cognitive dimensions of fatigue. Higher scores reflect greater severity of fatigue symptoms [22]. For the purposes of the present study, the combined total score of the two subscales was used in the primary analysis. Cognitive performance was assessed with the written version of the Symbol Digit Modalities Test (SDMT) [23], with higher scores reflecting better performance in information processing speed and sustained attention. Finally, depression was measured using the Hospital Anxiety and Depression Scale-Depression subscale (HADS-D), where higher scores indicated greater severity of depression symptoms [24].

### 2.4. Data Collection Procedure

Participants were recruited consecutively during their routine clinical visits at the included clinical centers. Written informed consent was obtained from all participants prior to enrollment, following a thorough explanation of the study aims, procedures, and confidentiality measures. Participation was voluntary, and all patients were informed of their right to withdraw at any time, without any consequences for their medical and clinical care. To ensure data accuracy, all information was initially documented on paper case report forms and subsequently transferred into a secure electronic database. Data entry procedures included double-checking to minimize transcription errors.

The study protocol was reviewed and approved by the Ethics Board Committee of Attikon University Hospital (ΕΒΔ48/23-01-24). The conduct and reporting of the study adhered to the Strengthening the Reporting of Observational Studies in Epidemiology (STROBE) guidelines (Supplementary Table S1) [25].

### 2.5. Statistical Analysis

Descriptive statistics were used to summarize variables (mean ± SD for continuous variables; frequency and percentage for categorical variables). The normality of all continuous variables was assessed through visual inspection of Q-Q plots and was found to be met.

For the primary analysis, employment status (0 = not employed, 1 = employed) was analyzed as a binary outcome using binary logistic regression (Enter method), with two-tailed tests and an alpha level of 0.05. Complete data were available for all variables included in the regression; therefore, the full sample (*N* = 78) was retained in the multivariable analyses. Prior to modeling, predictors were screened to minimize estimation problems by examining potential separation through cross-tabulations between categorical predictors and employment status and by confirming distributional overlap for continuous predictors across employment groups; no evidence of complete separation was identified. Multicollinearity was evaluated using Pearson correlations and collinearity diagnostics (tolerance and VIF) derived from an auxiliary linear regression including the same predictor set, indicating no problematic collinearity.

In the first block, demographic variables (sex, age, education level, and relationship status) were entered. In the second block, disease-related and symptom burden indices (disease duration, disability status as indexed by EDSS, and fatigue as indexed by MFIS) were added. In the third block, the presence of LUTS (yes/no) was entered. In the fourth block, cognitive performance was introduced using the SDMT score, and in the final block depressive symptoms were entered using the HADS-D. Model calibration was evaluated using the Hosmer–Lemeshow goodness-of-fit test, which supported adequate calibration (χ^2^(8) = 11.26, *p* = 0.187) [26].

Receiver operating characteristic (ROC) analysis was also conducted descriptively to summarize apparent discrimination based on the model-predicted probabilities for employment status (AUC with 95% confidence intervals; test against AUC = 0.50) [26]. Threshold analysis is reported in the Supplementary Material for exploratory purposes.

All statistical tests were performed using IBM SPSS Statistics (version 30.0) [27].

## 3. Results

### 3.1. Demographic Characteristics

A total of 89 pwMS were initially eligible for participation in the study, of whom nine declined due to personal reasons. The final sample comprised 78 participants. Of these, 57.7% were female (*n* = 45) and 42.3% (*n* = 33) were male, with a total mean age of 41.03 ± 10.45 years. Participants were treated with a variety of DMTs, most commonly ocrelizumab (16.7% of patients), followed by natalizumab (12.8%), dimethyl fumarate (11.5%), and ofatumumab (6.4%). In terms of relationship and occupational characteristics, 55.1% (*n* = 43) of participants reported being in a stable long-term relationship, and 52.6% (*n* = 41) were employed full-time. Regarding urinary functioning, 47.4% of participants (*n* = 37) reported the presence of LUTS, whereas 52.6% (*n* = 41) reported no LUTS. The mean disease duration was 8.9 ± 7.02 years, and the mean level of disability, as assessed by the EDSS, was 2.51 ± 1.02. With respect to cognitive status, the mean written SDMT score was 42.14 ± 10.74. Depression levels, as measured by the HADS-D, showed a mean score of 7.99 ± 3.67. Regarding fatigue, the mean total MFIS score was 32.31 ± 16.58 (see also Table 1 for descriptive statistics stratified by employment status).

### 3.2. Prediction of Employment Status and Model Discrimination

A hierarchical binary logistic regression was conducted to examine whether demographic, disease-related, clinical, cognitive, and psychological variables predicted employment status.

In Step 1, sex, age, education level, and relationship status were entered. This model did not reach statistical significance, χ^2^(4) = 8.61, *p* = 0.072, explaining 10.5% (Cox & Snell R^2^) to 13.9% (Nagelkerke R^2^) of the variance. Although the employed group included a higher proportion of single participants, relationship status was not an independent predictor of employment in the multivariable model.

In Step 2, disease duration, disability status (EDSS), and fatigue (MFIS) were added, which significantly improved model fit, Δχ^2^(3) = 14.40, *p* = 0.002. The Step 2 model was significant, χ^2^(7) = 23.01, *p* = 0.002, with Cox & Snell R^2^ = 0.255 and Nagelkerke R^2^ = 0.341. In this step, higher disability (EDSS) was associated with lower odds of being employed (OR = 0.33, *p* = 0.005), whereas fatigue and disease duration were not significant independent predictors (*p* > 0.05).

In Step 3, the presence of LUTS was entered and further improved the model, Δχ^2^(1) = 6.38, *p* = 0.012. The model was significant, χ^2^(8) = 29.38, *p* < 0.001, explaining 31.4% (Cox & Snell R^2^) to 41.9% (Nagelkerke R^2^) of the variance. The Hosmer–Lemeshow test did not indicate evidence of lack of fit, χ^2^(8) = 12.07, *p* = 0.148. In this step, LUTS presence was associated with lower odds of employment (OR = 0.14, *p* = 0.017), while EDSS was no longer significant (*p* > 0.05).

In Step 4, information processing speed (SDMT) was added and significantly improved fit, Δχ^2^(1) = 7.98, *p* = 0.005. The model was significant, χ^2^(9) = 37.36, *p* < 0.001, with Cox & Snell R^2^ = 0.381 and Nagelkerke R^2^ = 0.508. The Hosmer–Lemeshow test did not indicate evidence of lack of fit, χ^2^(8) = 12.71, *p* = 0.122. In this model, both LUTS presence (OR = 0.12, *p* = 0.023) and higher SDMT scores (OR = 1.10, *p* = 0.009) were significantly associated with employment status.

Finally, in Step 5, depressive symptoms (HADS) were entered but did not significantly improve model fit, Δχ^2^(1) = 0.57, *p* = 0.452. The final model remained significant overall, χ^2^(10) = 37.93, *p* < 0.001, explaining 38.5% (Cox & Snell R^2^) to 51.4% (Nagelkerke R^2^) of the variance. The Hosmer–Lemeshow test did not indicate evidence of lack of fit, χ^2^(8) = 11.26, *p* = 0.187. In the final model, LUTS presence (OR = 0.13, *p* = 0.026) and SDMT performance (OR = 1.11, *p* = 0.008) remained significantly associated with employment status, whereas disability (EDSS), fatigue (MFIS), depressive symptoms, and demographic/disease-duration variables were not significant (*p* > 0.05) (Table 2). To examine coefficient stability, internal validation was performed using bootstrap resampling (1000 iterations), indicating that the associations of SDMT and LUTS with employment status remained statistically supported across resamples (Supplementary Table S2).

In addition, ROC analysis based on model-predicted probabilities was conducted descriptively. Detailed threshold analyses are provided in the Supplementary Material (Supplementary Figure S1).

## 4. Discussion

The present study aimed to examine the extent to which commonly investigated demographic (sex, age, education, relationship status), disease-related (disease duration, fatigue), clinical (disability status), cognitive (information processing speed), and psychological (depressive symptoms) factors, alongside the presence of LUTS, predict employment status in pwMS. Overall, the findings indicated that information processing speed and LUTS status emerged as significant predictors of employment. Specifically, higher SDMT performance was associated with higher odds of being employed, whereas the presence of LUTS was associated with lower odds of employment. In contrast, disability status, fatigue, depressive symptoms, and demographic and disease-duration variables did not reach statistical significance once LUTS and processing speed were considered. ROC analysis provided a descriptive summary of discrimination within the present sample (AUC = 0.874). These findings should be interpreted cautiously given the lack of external validation.

Physical disability has consistently been linked with reduced work participation in MS [28,29,30], particularly as ambulation and motor endurance become compromised, and the findings of the present study partly align with this evidence. In their literature review, Pompeii and colleagues reported that unemployment was more common among pwMS who had greater disability, with individuals in the moderate EDSS range (approximately 3.0–6.0) being more likely to be unemployed than those with lower EDSS scores (<2.5–3.0), and the highest unemployment rates observed in those with EDSS scores of 6.5 or above [31]. Nevertheless, physical disability alone may not fully account for employment outcomes in MS. Although EDSS is a robust correlate of work ability, large registry data indicate that a substantial minority of pwMS discontinue working even at low EDSS levels, when physical impairment is minimal or absent, indicating that non-motor factors beyond overt disability may play a substantial role in shaping employment status [30]. This point may be particularly relevant to the present sample, which was characterized by relatively low disability [32]. In such cohorts, variability in work participation may be less strongly driven by overt physical limitation and may be shaped to a greater extent by other determinants of day-to-day occupational functioning. This is in line with our results, as EDSS was associated with employment status in earlier blocks of the model; however, when additional MS-related domains were considered, EDSS no longer emerged as a correlate. This attenuation does not necessarily indicate that physical disability is irrelevant to occupational status; instead, it may reflect shared variance and/or mediation pathways, especially given the relatively low EDSS range in this cohort.

Consistent with the above view, the literature indicates that cognitive functioning plays a central role in work outcomes in MS, with difficulties ranging from basic domains such as attention and information processing speed to higher-order abilities including learning and memory, cognitive flexibility, problem-solving, and organization [8,33,34]. In the present study, information processing speed was selected as a representative cognitive marker because it is the most frequently affected domain in MS and is thought to exert downstream effects on more complex cognitive operations [35,36]. This focus may also be highly relevant to modern occupational demands: across many workplaces, efficiency under time pressure, multitasking, and rapid information handling are essential [37], and reduced processing speed may manifest as slower task completion, increased errors, and greater perceived effort. Over time, these challenges may necessitate workplace accommodations and can contribute to work instability or job loss, underscoring processing speed as a meaningful target when examining employment status in MS. Consistent with prior work [30,38,39,40], our results further support the notion that processing speed contributes uniquely to employment status in pwMS, even when accounting for other commonly examined factors.

It is important to note that although employment status has been consistently associated with lower fatigue levels [32,33,39,41], it did not emerge as a significant predictor in the present multivariable model. One plausible explanation is that the association between fatigue and employment may be partly accounted for by disability status and cognitive efficiency, which are themselves closely linked to fatigue severity and may attenuate its unique contribution once entered simultaneously [42,43]. In particular, reduced information processing speed may increase cognitive load and the effort required to complete everyday tasks, contributing to earlier mental exhaustion and higher perceived cognitive and physical fatigue [44]. At the same time, evidence also supports the reverse direction, whereby higher fatigue levels, especially in progressive MS, are associated with poorer processing speed, suggesting a bidirectional relationship [45,46,47]. Taken together, these findings point to a potentially self-reinforcing cycle in which neurodegenerative processes contribute to cognitive slowing; slower cognition increases the time and effort required for routine activities, and the resulting fatigue further compromises key functions such as processing speed, attention, concentration, and memory.

This cognitive–fatigue loop likely represents only one pathway, as fatigue in MS is inherently multifactorial, with contributors that often overlap with depressive and anxiety symptoms as well as sleep disturbance [48], and its functional impact may be attenuated in some individuals through compensatory approaches, workplace adjustments, or flexible scheduling. In a similar way, mood-related symptoms can undermine work participation by reducing motivation, impairing concentration, and lowering stress tolerance, thereby affecting both performance and endurance at work [40,49]. Beyond symptom burden, a number of sociodemographic and disease-related factors have also been linked to unemployment in pwMS, including lower educational attainment, older age, longer disease duration, a progressive disease course, and greater symptom severity at onset. Evidence further suggests that the risk of work disability is elevated among older individuals and those experiencing work instability, and that part-time employment may represent a marker of vulnerability to subsequent work loss [41]. Sex-related differences have also been reported, with some studies indicating higher risk of labor-market withdrawal and work absence among women [50]; the risk of work “drift” has been estimated to be substantially higher in females [51]. Nevertheless, the extent to which these variables exert independent effects is not uniform across studies and likely depends on sample characteristics (e.g., disability range, disease phenotype), measurement approaches, and whether key correlates are entered concurrently in multivariable models, which can attenuate or eliminate associations that appear robust in univariable analyses.

Importantly, urinary dysfunction is a common and clinically meaningful manifestation of MS, affecting up to 80% of pwMS, and is typically associated with increasing age, longer disease duration, and a more progressive disease course [15,52]. In the present study, LUTS were associated with employment status, raising the possibility that bladder-related difficulties may represent an additional, and often overlooked, factor related to work participation. From an occupational perspective, symptom unpredictability and the need for frequent or urgent bathroom access may disrupt sustained attention, task continuity, commuting, and participation in work tasks with limited flexibility, potentially increasing the likelihood of workplace strain, reduced productivity, or eventual withdrawal from employment. Indicators related to incomplete bladder emptying, such as post-void residual (PVR) volume, may reflect clinically significant neurogenic bladder involvement and may serve as objective markers of symptom burden [53,54]. In our dataset, LUTS were captured via dichotomous routine clinical self-report without standardized symptom quantification. Moreover, information on symptom duration, urological comorbidities, and current urological treatments was not available. Therefore, LUTS in the present sample may partially reflect disease burden, comorbidity, or contextual factors not captured in the model, and the observed association with employment status should be interpreted cautiously. Nevertheless, given that bladder dysfunction remains underrecognized and undertreated in clinical practice [55], these findings may highlight the need for future studies to integrate this domain into assessment and monitoring, helping identify individuals who could benefit from earlier targeted interventions.

From a pragmatic standpoint, combining SDMT performance with LUTS status may help clinicians identify individuals who, in this sample, are more likely to be non-employed, and may inform more general clinical conversations regarding occupational challenges. However, up to this point, any use for prognostication or forecasting future work outcomes will require validation in independent and preferably longitudinal cohorts to further support these claims. Nevertheless, to our knowledge, this study is among the first to complement multivariable prediction of employment status in pwMS with an evaluation of discrimination performance (ROC/AUC), thereby providing an initial indication of the potential clinical utility of integrating a routinely assessed cognitive marker together with an often under-recognized symptom domain for flagging individuals who might benefit from earlier monitoring, counseling, and supportive interventions.

These findings have plausible clinical relevance, as both urinary dysfunction and reduced processing speed may translate into greater perceived effort, reduced efficiency, and lower tolerance for complex or time-pressured work demands, thereby increasing vulnerability to workplace strain, the need for job modification or reduced responsibilities, and, in some cases, eventual withdrawal from employment. Accordingly, clinicians may consider remaining attentive to early signs of difficulty in these domains and initiating proactive discussions about potential workplace challenges. Where appropriate, timely workplace adjustments and symptom-focused management may be encouraged, alongside early referral to relevant services (e.g., urology or continence services, vocational rehabilitation, occupational therapy, cognitive rehabilitation, and psychosocial support) to help support sustained employment and prevent avoidable work loss.

### Limitations and Future Research

Several limitations should be considered when interpreting these findings. First, because this was a cross-sectional study, the model probabilities should be interpreted as classification of current employment status rather than prediction of future outcomes; longitudinal validation is required before the model can be used prognostically. Second, the relatively limited sample size may raise the possibility of statistical overfitting, possibly limiting the stability and generalizability of the estimates. It is important for future studies to replicate these results in larger cohorts and validate the model. Third, employment was operationalized as a binary outcome, with part-time workers excluded to maintain a clear dichotomization. Future research should examine more granular employment outcomes (e.g., part-time work, work instability, sick leave, presenteeism) to better reflect real-world occupational functioning in MS. Fourth, LUTS was assessed using a dichotomous presence/absence indicator rather than a standardized severity measure, which may introduce misclassification due to variability in symptom inquiry and reporting, comorbid urological conditions, or treatment effects. Thus, future studies should incorporate standardized questionnaires and/or objective indices of LUTS severity, frequency, and functional impact to clarify the dose–response relationship with employment outcomes. Finally, cognition was represented primarily by processing speed; although clinically meaningful, broader cognitive profiling (e.g., executive functions and memory) and contextual factors (e.g., fatigue management strategies, mental health history, socioeconomic variables, and workplace characteristics) should be incorporated in future models to clarify mechanisms and improve predictive accuracy. The results and how they can be interpreted from the perspective of previous studies and of the working hypotheses should be discussed. The findings and their implications should be discussed in the broadest context possible. Importantly, ROC/AUC estimates and probability cut-offs were derived from the same dataset used to fit the model; therefore, these indices and thresholds should be interpreted as exploratory and hypothesis-generating and should be replicated and validated in larger cohorts with internal and external validation and calibration assessment.

Employment participation in MS reflects a highly multidetermined and context-dependent outcome, arising from multiple determinants. Accordingly, the present findings should be interpreted in light of the restricted covariate set available for modeling. Several potentially important determinants of employment were not captured, including MS phenotype and disease course, markers of recent disease activity (e.g., relapses), detailed treatment characteristics (DMT class, adverse effects, and concomitant medications), and key contextual factors such as type of occupation (physical vs. cognitive demands), schedule flexibility or remote-work options, workplace accommodations, household income and disability benefits, and more granular employment categories (e.g., retirement, temporary leave, or disability pension). The absence of these variables introduces the possibility of residual confounding and limits the extent to which the model can be considered comprehensive and complete. For this reason, our conclusions are restricted to the variables included in the present analysis, and LUTS and SDMT are best viewed as variables that remained significantly associated with employment status within this set rather than as definitive determinants of employment outcomes.

## 5. Conclusions

Τhis multicenter cross-sectional study pointed out that among a range of commonly examined demographic, disease-related, clinical, cognitive, and psychological factors, information processing speed and the presence of LUTS emerged as the independent correlates of full-time employment in pwMS, suggesting that less visible, but functionally impactful, symptom domains may play a key role in work participation, particularly in cohorts with relatively low levels of physical disability. Within the present sample, the LUTS–SDMT specification provided a descriptive summary of discrimination between employed and non-employed participants. However, these findings should be interpreted as exploratory and they require confirmation in independent cohorts. Future research should examine these associations longitudinally and evaluate model performance using external validation.

## Figures and Tables

**Table 1:** Participant characteristics by employment status (*N* = 78).

Variables	Non-Employed (*n* = 37)		Employed (*n* = 41)	
	M (SD)/*n* (%)	95% CI	M (SD)/*n* (%)	95% CI
Sex				
Female, *n* (%)	22 (59.5%)	-	23 (56.1%)	-
Male, *n* (%)	15 (40.5%)	-	18 (43.9%)	-
Relationship status				
Single, *n* (%)	11 (29.7%)	-	24 (58.5%)	-
In relationship/Married, *n* (%)	26 (70.3%)	-	17 (41.5%)	-
LUTS				
Yes	28 (75.7%)	-	9 (22%)	-
No	9 (24.3%)	-	32 (78%)	-
Age (years)	43.76 (10.52)	40.25, 47.27	38.56 (9.88)	35.44, 41.68
Education level (years)	14.22 (2.04)	13.54, 14.90	14.85 (2.06)	14.20, 15.50
Disease duration (years)	10.46 (7.34)	8.01, 12.91	7.49 (6.48)	5.44, 9.53
EDSS	2.87 (1.00)	2.53, 3.21	2.08 (0.86)	1.81, 2.36
SDMT (written)	37.78 (10.13)	34.41, 41.16	46.07 (9.81)	42.98, 49.17
MFIS total	37.62 (15.59)	32.42, 42.82	27.51 (16.14)	22.42, 32.61
HADS–D	8.41 (3.68)	7.18, 9.63	7.61 (3.66)	6.45, 8.76

Note: Values are presented as M (SD) for continuous variables and *n* (%) for categorical variables; 95% confidence intervals (CI) are shown for continuous variables. EDSS = Expanded Disability Status Scale; HADS–D = Hospital Anxiety and Depression Scale-Depression subscale; LUTS = Lower Urinary Tract Symptoms; MFIS = Modified Fatigue Impact Scale; SDMT = Symbol Digit Modalities Test.

**Table 2:** Hierarchical binary logistic regression predicting employment status: Final model (Step 5).

Predictors	B	SE	Wald	*p*	OR	95% CI for OR
Sex	−0.14	0.63	0.05	0.824	0.87	[0.25, 2.99]
Age	−0.02	0.04	0.18	0.670	0.98	[0.90, 1.07]
Relationship status (Single vs. In a relationship)	−1.27	0.81	2.43	0.119	0.28	[0.06, 1.39]
Education level	−0.01	0.16	0.01	0.947	0.99	[0.72, 1.36]
Disease duration	0.06	0.06	1.12	0.289	1.06	[0.95, 1.19]
EDSS	−0.67	0.53	1.59	0.207	0.51	[0.18, 1.45]
MFIS	0.05	0.03	2.16	0.142	1.05	[0.98, 1.12]
LUTS	−2.06	0.93	4.93	0.026	0.13	[0.02, 0.79]
SDMT	0.10	0.04	6.97	0.008	1.11	[1.03, 1.19]
HADS-D	−0.07	0.09	0.56	0.456	0.93	[0.78, 1.12]

Note: Outcome coding: 0 = not employed, 1 = employed. CI = confidence interval; EDSS = Expanded Disability Status Scale; HADS-D = Hospital Anxiety and Depression Scale; LUTS = Lower Urinary Tract Symptoms; MFIS = Modified Fatigue Impact Scale; OR = odds ratio; SDMT = Symbol Digit Modalities Test. Model fit: χ^2^(10) = 37.93, *p* < 0.001; −2LL = 69.99; Cox & Snell R^2^ = 0.385; Nagelkerke R^2^ = 0.514; Hosmer–Lemeshow χ^2^(8) = 11.26, *p* = 0.187. Classification accuracy = 78.2%.

## Data Availability

The data are available on request from the corresponding author. The data are not publicly available due to ethical and privacy restrictions.

## Supplementary Materials

The following supporting information can be downloaded at https://www.mdpi.com/article/10.3390/medicina62030454/s1; Table S1: STROBE Statement—checklist of items that should be included in reports of observational studies; Table S2: Bootstrap Internal Validation of the Final Hierarchical Logistic Regression Model (Step 5); Figure S1: Receiver operating characteristic (ROC) curve for the logistic regression model predicting employment status.
